# Development of Molecular Markers for Iron Metabolism Related Genes in Lentil and Their Expression Analysis under Excess Iron Stress

**DOI:** 10.3389/fpls.2017.00579

**Published:** 2017-04-13

**Authors:** Debjyoti Sen Gupta, Kevin McPhee, Shiv Kumar

**Affiliations:** ^1^Department of Plant Sciences, North Dakota State University, FargoND, USA; ^2^Division of Crop Improvement, ICAR-Indian Institute of Pulses ResearchKanpur, India; ^3^BIGM Program, International Center for Agricultural Research in the Dry Areas (ICARDA), Rabat-InstitutesRabat, Morocco

**Keywords:** lentil, gene expression, iron metabolism, qPCR expression analysis, molecular marker, ferritin

## Abstract

Multiple genes and transcription factors are involved in the uptake and translocation of iron in plants from soil. The sequence information about iron uptake and translocation related genes is largely unknown in lentil (*Lens culinaris* Medik.). This study was designed to develop iron metabolism related molecular markers for *Ferritin-1, BHLH-1* (Basic helix loop helix), or FER-like transcription factor protein and *IRT-1* (Iron related transporter) genes using genome synteny with barrel medic (*Medicago truncatula*). The second objective of this study was to analyze differential gene expression under excess iron over time (2 h, 8 h, 24 h). Specific molecular markers were developed for iron metabolism related genes (*Ferritin-1, BHLH-1, IRT-1*) and validated in lentil. Gene specific markers for *Ferritin-1* and *IRT-1* were used for quantitative PCR (qPCR) studies based on their amplification efficiency. Significant differential expression of *Ferritin-1* and *IRT-1* was observed under excess iron conditions through qPCR based gene expression analysis. Regulation of iron uptake and translocation in lentil needs further characterization. Greater emphasis should be given to development of conditions simulating field conditions under external iron supply and considering adult plant physiology.

## Introduction

Iron (Fe) uptake in plants is a complex physiological process governed by homeostatic mechanisms in the plant. Homeostatic mechanisms involve absorption, translocation and redistribution of Fe within the plant system at a particular concentration (10^-9^–10^-4^ mol/l) (Römheld and Schaaf, 2004). Lower iron concentration leads to Fe-deficiency symptoms including chlorosis and necrosis in leaves and ultimately loss in biomass as well as grain yield. Higher concentrations of Fe within the plant system results in generation of free radical species which damage various cellular components by interacting with protein, lipid, carbohydrates and even with DNA. According to Welch and Graham (2004), there are four different barriers controlling homeostatic mechanisms of mineral uptake in plants; (A) the root-soil interphase known as the rhizosphere, (B) root-cell plasma membrane, (C) translocation to edible plant organs (grains/tubers), and (D) bioavailability of minerals.

Ferritin is an iron-carrying protein in plants and has a multimeric (24-mer) cage-like structure that carries up to 4500 atoms of Fe within its core (Crichton et al., 1978; Wade et al., 1993). The ferritin protein is highly conserved within the animal and plant kingdom (Ragland et al., 1990). Ferritin meets the metabolic need for iron when required by the metabolome as well as prevents any kind of oxidative stress (Harrison et al., 1998). Plant ferritin subunit sequences share between 39 and 49% similarity with mammalian ferritin sequences (Briat et al., 2009). This similarity increases when comparisons are made within the plant kingdom or among close plant families. Iron homeostasis is important due to the minute balance that exists between iron deficiency and toxicity and that affects plant physiology. Impaired plant physiology ultimately affects crop yield. Ferritin regulates iron homeostasis to prevent interaction of iron with other cellular components which may result in generation of free radicals during oxidative stress. In plants, ferritin consists of a single kind of subunit and ferritin bound Fe is highly bioavailable (Kalgaonkar and Lonnerdal, 2008).

Lentil (*Lens culinaris* Medik.) is a eudicot plant and uses strategy I where ferric iron is reduced at the rhizosphere and absorbed as ferrous iron by the root. Monocot plants use a different strategy to uptake iron from the soil (strategy II). The uptake of iron is mediated through phytosiderophores and the ferric iron enters the plant system through root in case of monocot plants (strategy II). In *Arabidopsis thaliana*, reduction of ferric Fe is accomplished by Fe reductase FRO2 (ferric reductase oxidase-2; Robinson et al., 1999). This was the first report of cloning and gene function elucidation of any major iron metabolism related gene in plants. Uptake of ferrous Fe into the root is carried out by the metal transporter IRT1 (iron-regulated transporter; Eide et al., 1996; Vert et al., 2002). The basic helix-loop-helix (BHLH) transcription factor family in plants is a ubiquitous regulator and is highly conserved, regulating different types of genes during transcription (Heim et al., 2003). The BHLH transcription factor or FIT (FER-like Fe deficiency-induced transcription factor) is reported to be responsible for high-level expression of FRO2 and IRT-1 (Colangelo and Guerinot, 2004; Jakoby et al., 2004; Yuan et al., 2005). It is pertinent to mention that iron uptake, translocation and storage is a complex pathway and multiple genes or gene families are involved. However, from crop breeding point of view breeders always need a high-throughput and less time consuming techniques to identify few potential genotypes in a large set of germplasms. These three genes were targeted in lentil with a long-term objective in mind to develop an assay to find lentil genotypes which better perform under excess iron supply. The results of this experiment would give an initial thrust to such objectivities in lentil where limited amount of sequence information is available till date.

Development of gene specific markers and their utilization in understanding metabolic pathways are important genomic goals to achieve in any crop species for their effective utilization in genetic studies or molecular breeding applications *per se*. Availability of specific DNA markers for iron metabolism related genes in lentil are not available. The objectives of the study were to, (1) develop gene (*Ferritin-1, BHLH-1*, and *IRT-1*) specific molecular markers in lentil and (2) analyze their gene expression under excess iron over time.

## Materials and Methods

### Plant Materials and Treatments

‘CDC Redberry’ (Vandenberg et al., 2006) seedlings were raised in the laboratory and fresh tissue was collected for DNA and RNA extraction. Seeds were germinated on wet filter paper in an incubator maintained at 25°C. Seedlings were transferred to 50 mL tubes containing distilled water for hydroponic growth with 16:8 h light: dark cycle and at 25°C for eight days after germination. After complete development of the first true leaf (of growth), treatments were applied 18–21 days after germination and included: (1) iron deficient condition (control with distilled water), (2) excess iron condition (addition of 500 μM of Fe-EDTA, 150 mM of sodium citrate, and 75 μM FeSO_4_) (Lobréaux et al., 1995). Treatments were applied for 24 h and samples were collected 2, 8, and 24 h after treatment. Three biological replications were included for each treatment.

### Development of Markers

Full length coding sequences (CDS) for three ferritin genes (*ferritin-1, ferritin-2, ferritin-3*) for *Medicago truncatula* were acquired from the NCBI (National Center for Biological Information) nucleotide database on 15 April 2015. The complete coding sequence of *Ferritin-2* mRNA (NCBI reference sequence: XM_003616637.1) of *M. truncatula* was downloaded in FASTA format and used to perform a nucleotide BLAST search against CDC Redberry 454 contig sequences in the Knowpulse database^1^. The contig sequence with the highest bit score and lowest e-value and, therefore, having the highest similarity with the query sequence (*M. truncatula Ferritin-2*) was identified. The contig sequence was downloaded from the Knowpulse database and Primer-BLAST^2^ was used to design primer pairs using default parameters (**Table 1**). One primer pair (FerrClo5) used for the development of qPCR compatible primers for the *Ferritin* gene in lentil. In addition, one primer pair specific to a lentil BHLH (Basic Helix Loop Helix) transcription factor or FER-like transcription factor gene sequence (Sen Gupta et al., 2016) was synthesized. Primers were also designed for the iron-related transporter gene based on the *IRT1* mRNA coding sequence (CDS) (LegumeIP database reference no. IMGA[Medtr8g105030.1] of *M. truncatula* for the amplification of lentil *IRT-1* in the qPCR experiment. The amplicon of the ferritin gene as well as the BHLH transcription factor gene were beyond the range of optimum product size (>250 bp) for qPCR experiments and thus were gel purified using a gel purification kit (IBI, MIDSCI, St. Louis, MO, USA) (Vogelstein and Gillespie, 1979) following manufacturer’s instructions and sequenced using the Sanger sequencing method (Etonbiosciences Inc., San Diego, CA, USA). The gene sequences were aligned with the respective *M. truncatula* mRNA sequences (*Ferritin-2* and *BHLH* transcription factor gene, respectively) and primers were designed for qPCR experiments based on the putative exonic sequences, their sequence identity, gap, and the desired product size using Primer3 software^2^. Based on these sequences one primer pair for *Ferritin-1* and another primer pair for the *BHLH-1* transcription factor were designed for qPCR. Primers for *IRT-1* were directly used in qPCR and were within the qPCR compatible product size range (<100 bp amplicon size).

**Table 1:** Nucleotide BLAST results of *Medicago truncatula ferritin-2* gene sequence (NCBI reference no. XM_003616637.1) with CDC Redberry 454 contig sequences in Knowpulse database showing bit score, percent identity, and *e*-value (http://knowpulse.usask.ca).

Hit^∗^	Bit score	Identity%	*E*-value
LcRBContig00605	700	91	0.00e+0
LcRBContig02360	530	90	1.53e-103
LcRBContig20139	142	93	4.44e-5
LcRBContig24460	167	94	1.39e-40
LcRBContig24460	167	94	1.39e-40
LcRBContig13391	167	94	1.39e-40
LcRBContig07868	167	94	1.39e-40
LcRBContig07177	167	94	1.39e-40
LcRBContig01318	167	94	1.39e-40
LcRBContig24151	111	91	7.13e-24

*^∗^First 10 relevant hits are shown here.*

### Isolation of RNA and Synthesis of Complementary DNA

Total RNA was extracted from 100 mg of fresh leaves of individual treatments using the QIAGEN^®^ RNeasy Mini Kit (QIAGEN, Valencia, CA, USA) according to manufacturer instructions. The quality of the RNA extracts were determined by the spectrophotometer Nano-Drop (ND-1000) (NanoDrop Technologies, Welmington, DE, USA). To check the integrity of the RNA, the samples were stained, separated and visualized by electrophoresis in a 1% agarose gel. Details about the quality of the RNA samples can be found in Supplementary Table S1. The first strand of cDNA was synthesized from 1 μg of total RNA in a 20 μL reaction using SuperScript III First Strand Synthesis Supermix RT-PCR Kit (Invitrogen, USA). The cDNAs were diluted to 2 ng μL^-1^.

### Quantitative PCR

Three primer pairs were used for gene expression analysis, *Ferritin1* (developed using PCR based cloning and sequencing), *BHLH1* (developed using PCR based cloning and sequencing) and *IRT1* (primer designed based on *M. truncatula IRT1* gene sequence). Expression levels of mRNA were evaluated in a SYBR Green dye using an Applied Biosystems 7500 Fast Real-Time PCR System (Applied Biosystems, USA). PCR amplifications were carried out in triplicate in 20 μL reactions containing Maxima SYBR Green mixer (Fermentas, USA), 250 nM of each primer and 4 ng of cDNA. On each plate, the reference genes (*GADPH* and *Actin*) and negative controls were included. Amplification conditions were 50°C for 2 min, 95°C for 10 min, 40 cycles at 95°C for 15 s, 60°C for 1 min. The calibration curves for each primer pair were plotted using five serial dilutions of the cDNA in water. To verify the specificity of amplification a dissociation curve analysis step was added to the qPCR amplification protocol. Amplification efficiency, slope and R^2^ value were determined for each primer pair. Amplification efficiencies were calculated as *E* = (10^-1/slope^–1) × 100.

### Statistical Analysis of Gene Expression Analysis

Cycle threshold (*C*_T_) values were determined using SDS software (Applied Biosystems, USA). Gene expression data were analyzed using the C_T_ values and amplification efficiency values using method 2^-ΔΔCT^ (Livak and Schmittgen, 2001). Geometric means of reference genes were used to normalize the C_T_ values of the individual samples. The program REST 2009—Relative Expression Software Tool (Pfaffl, 2001) was used to determine if the differences between the treatments were statistically significant (*P* < 0.05).

## Results

### Development of Markers for *Ferritin-1, BHLH-1*, and *IRT-1* Genes

**FIGURE 1:**
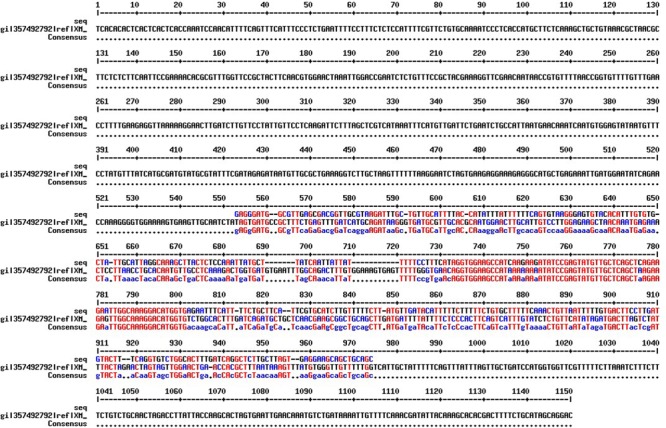
**Sequence alignment between *Medicago truncatula ferritin-2* full length CDS (NCBI reference no. XM_003616637.1) and lentil *Ferritin-1* partial genomic sequence using the MultAlin tool (Corpet, 1988) with default parameter values.** The overlapping potential exonic region (95 bp) is marked in blue and red color.

After performing BLASTn analysis using *ferritin-2* mRNA sequence of *Medicago truncatula* in the KnowPulse database (University of Saskatchewan, Canada) one contig sequence was identified, LcRBContig00605, based on BIT score (700), sequence identity (91%), and *e*-value (0) (**Table 1**). BLASTn results using other plant species resulted in identification of this contig sequence (LcRBContig00605) (data not shown). Optimum PCR conditions for the designed primers (FerrClo5) in an ABI 7500 thermocylcer were established: 94°C for 5 minutes, followed by 30 cycles of 94°C for 1 min, 60°C for 1 m, 72°C for 1 min followed by a final elongation step of 72°C for 5 min. The amplified DNA fragment was gel purified and sequenced using Sanger’s method to obtain a 390 bp sequence. Alignment of the partial genomic DNA sequence with the *M. truncatula ferritin-2* mRNA sequence (NCBI reference sequence: XM_003616637.1) showed a 92 bp sequence overlap with no gap (**Figure 1**). This potential exonic sequence was used to design primers (*Ferritin-1*) using Primer-BLAST (**Table 2**).

**Table 2:** Sequence and *T*_m_ (melting temperature) for primers designed based on the CDC Redberry contig (LcRBContig00605) for the *Ferritin-1* gene in lentil.

Primer name	Forward sequence (5′–3′)	Reverse sequence (5′–3′)	*T*_m_ (°C)
FerrClo1	TGCTGATAAGGGTGATGCGCT	GGCTTCCACCTGTTCACCCA	64
FerrClo2	AACCTGCACAGTGTTGCCTC	AGTGCCAGACACCATGTCCT	62
FerrClo3	GTTGCGCTGAAAGGTCTTGCT	GCCAAGTGCACATCACCAGT	62
FerrClo4	ACGTTGCGCTGAAAGGTCTTG	TGCCAAGTGCACATCACCAG	62
FerrClo5	CTGGTGATGTGCACTTGGCA	GCTGCAGCTGCTTCCTCACT	62

**FIGURE 2:**
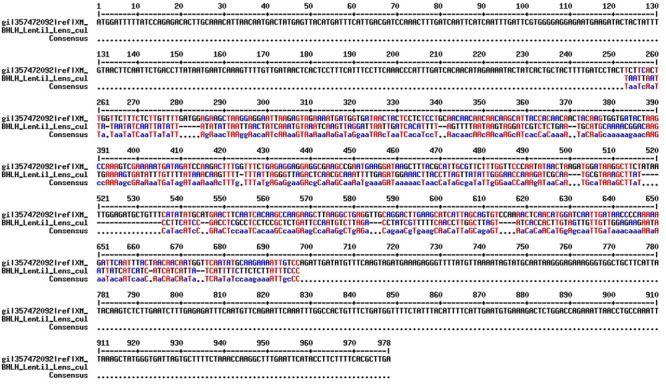
**Sequence alignment between *M. truncatula BHLH* full length CDS (NCBI reference number XM_003606283.1) and lentil *BHLH-1* partial genomic sequence using the MultAlin (Corpet, 1988) with default parameter values.** The overlapping potential exonic region (72 bp) is marked in blue and red color.

Primer pairs developed in a previous study (Sen Gupta et al., 2016) were used to amplify the *BHLH-1* gene in CDC Redberry genomic DNA. Optimum PCR conditions for *BHLH-1* primer pairs in an ABI 7500 thermocylcer were established: 94°C for 5 min, followed by 30 cycles of 94°C for 1 min, 60°C for 1 min, 72°C for 1 min followed by a final elongation step of 72°C for 5 min. The amplified fragment sequenced by Sanger’s sequencing method and A 490 bp sequence was obtained. This sequence was aligned with *M. truncatula BHLH* mRNA sequence (NCBI reference number XM_003606283.1) and based on the alignment (**Figure 2**) a 75 bp sequence with no gap (potential exonic sequence) was used to design qPCR compatible primers for *BHLH-1* in lentil using Primer-BLAST.

Using a *M. truncatula* iron regulated transporter gene mRNA sequence [LegumeIP database reference no. IMGA(Medtr8g105030.1)] primer pairs (IRT1) were designed for the qPCR study.

**FIGURE 3:**
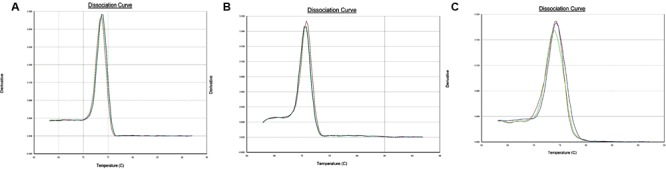
**(A)** Dissociation curve for *Ferritin-1* primer pairs. Derivative plotted in Y-axis is the negative of the rate of change in fluorescence as a fraction of temperature and temperature is plotted on the *X*-axis. **(B)** Dissociation curve for *BHLH-1* primer pairs. Derivative plotted in *Y*-axis is the negative of the rate of change in fluorescence as a fraction of temperature and temperature is plotted on the *X*-axis. **(C)**. Dissociation curve for *IRT-1* primer pairs. Derivative plotted in *Y*-axis is the negative of the rate of change in fluorescence as a fraction of temperature and temperature is plotted on the *X*-axis.

Dissociation curve analysis of the three pairs of primers (*Ferritin-1, BHLH-1, IRT-1*) showed specific amplification (**Figure 3**). Amplification efficiency of the designed primer pairs and reference genes (*GADPH, Actin*) were found to be >90% with the exception of *BHLH-1* primer pairs (**Table 3**). Slope values ranged from –0.02 to –3.55 and *R*^2^ values ranged between 0.0034 and 0.9972.

**Table 3:** Amplification statistics for one *Ferritin-1*, one *BHLH-1*, one *IRT-1* gene specific primer pairs, and one primer pair for each reference gene (*GADPH, Actin*).

Gene	Forward sequence	Reverse sequence	*T*_m_ (°C)	Size (bp)	Slope	*R*^2^	*E*	Reference
*Ferritin-1*	AGATATCCGAGTATGTTGCTCAG	AAGATGCACGAATGAAGCAGAAA	61	84	–3.32	0.9968	100.07	Current work
*IRT-1*	GTCGCTGTTTTGCTAGGTGC	GTGAGCTTCTCCTCTTCCCT	61	159	–3.12	0.9954	109.18	Current work
*BHLH-1*	TTATTAGGGTTAGACTCAACGCA	TTGCGATCTTTGGTTCCCA	59	74	–0.02	0.0034	6.55e+42	Sen Gupta et al., 2016
*GADPH*	TGGGCGAAAACTCCACTTTG	GAATTGCTGCAGCCTTGTGA	60	57	–3.15	0.9954	107.71	Saha and Vandemark, 2012
*Actin*	CCAAATCATGTTTGAGGCTTTTAA	GTGAAAGAACGGCCTGAATAGC	60	64	–3.55	0.9972	91.25	Saha and Vandemark, 2012

*Here, *T*_*m*_ = melting temperature, Size = amplicon length, Slope = slope of the trend line in amplification efficiency graph, *R*^*2*^= regression coefficient, *E* = amplification efficiency.*

### Expression Analysis of *Ferritin-1* and *IRT-1* Genes

Using the 2^-ΔΔCT^ method (Livak and Schmittgen, 2001), changes in gene transcripts were calculated for the treated samples (under excess iron condition) compared to the control treatments (iron-deficient condition) (**Table 4**). The changes in gene transcript levels for *Ferritin-1* and *IRT-1* genes were not significantly different for the shoot tissue (**Table 5**). A 2.72-fold increase in *Ferritin-1* gene transcripts was observed in root tissue after 2 h of iron treatment (*P* < 0.05) (**Table 5**). Similarly, a 3.6-fold increase in *IRT-1* gene transcripts was observed (*P* < 0.05) (**Table 5**).

**Table 4:** Differentially expressed *Ferritin-1* and *IRT-1* genes in CDC Redberry shoot and root tissues over time (2, 8 and 24 h) in three replicates under excess iron.

Gene	Plant tissue	Time course					
		2 h	2 h	2 h	8 h	8 h	8 h	24 h	24 h	24 h
	Shoot tissue									
*Ferritin-1*		0.29	1.0	3.03	0.47	0.79	1.45	2.7	1.41	0.83
*IRT-1*		0.37	1.47	0.38	0.15	0.44	1.79	1.38	1.0	0.20
	Root tissue									
*Ferritin-1*		1.81	3.29	3.05	0.22	0.52	1.45	0.64	0.32	0.82
*IRT-1*		1.70	5.03	4.59	0.30	0.55	1.73	0.73	0.44	1.14

**Table 5:** Significance of differential expression of samples over time (TC) in excess iron in relation to control samples in shoot and root tissue of CDC Redberry genotype.

TC (h)	Gene	Tissue	*N*	*E*	*SE*	95% CI	*P*(H1)	Remark
2 h	*Ferritin-1*	Shoot	3	0.474	0.241–0.938	0.159–1.264	0.199	NS
8 h	*Ferritin-1*	Shoot	3	1.056	0.763–1.411	0.711–1.843	0.724	NS
24 h	*Ferritin-1*	Shoot	3	1.049	0.589–2.150	0.377–2.792	0.832	NS
2 h	*Ferritin-1*	Root	3	2.724	1.866–4.644	1.342–5.267	0	Up regulated, significant
8 h	*Ferritin-1*	Root	3	0.554	0.310–1.018	0.228–1.395	0.28	NS
24 h	*Ferritin-1*	Root	3	0.558	0.383–0.796	0.096		NS
2 h	*IRT-1*	Shoot	3	0.591	0.223–1.443	0.116–2.883	0.539	NS
8 h	*IRT-1*	Shoot	3	0.487	0.218–1.634	0.162–1.835	0.298	NS
24 h	*IRT-1*	Shoot	3	0.65	0.283–1.280	0.211–2.734	0.517	NS
2 h	*IRT-1*	Root	3	3.563	2.186–5.066	1.874–5.405	0	Up regulated, significant
8 h	*IRT-1*	Root	3	0.672	0.386–1.101	0.313–1.640	0.245	NS

*Here, *N* = number of biological replications, *E* = Differentiial expression, *SE* = standard error, *P*(H1) = Probability of alternative hypothesis.*

## Discussion

Iron uptake from the soil and translocation within the plant is a complex physiological process. It involves multiple genes and transcription factors. The magnitude of mRNA transcript synthesis under excess iron conditions for iron metabolism related genes (*Ferritin-1, BHLH-1, IRT-1*) in lentil was evaluated in this study. Two genes, *Ferritin-1 and IRT-1*, were quantitatively assayed for differential gene expression as they exhibited amplification efficiency of >90 percent (Udvardi et al., 2008).

Dissociation curve analysis (**Figure 3**) which is the dsDNA melting curve analysis (Udvardi et al., 2008) added at the end of PCR run showed the specificity for single amplicon amplification and expected melting temperature for the individual primer pairs. All of the three primer pairs exhibited a typical single peak with expected melting temperatures (**Figure 3**). Gene expression quantification values (*C*_T_ values) were normalized using geometric means of *C*_T_ values of the two reference genes (*GADPH, Actin*) (Vandesompele et al., 2002). *Actin* and *GADPH* were used in studies in lentil, pea and common bean exhibiting stability of expression across tissues and plant parts (Saha and Vandemark, 2012, 2013, DeLaat et al., 2014). The objective behind the normalization of qPCR data was to remove the sampling error, which may arise due to RNA quantity and quality differences across samples.

In this study, we developed gene-specific molecular markers for three genes (*Ferritin-1, BHLH-1, IRT-1*) in lentil. Primers for *Ferritin-1*and *IRT-1* were used in differential gene expression analysis. Partial genomic DNA sequences of *Ferritin-1* and *BHLH-1* were submitted to the NCBI database. These sequences are available to clone full length genomic sequences of each gene in lentil. The partial genomic DNA sequence *BHLH-1* gene can be further analyzed and used to develop qPCR compatible primers for this gene. It can be hypothesized from the comparative genomic synteny of lentil with *M. truncatula* (Phan et al., 2007) that a ferritin gene family does exist in lentil and other ferritin genes in *M. truncatula* (*ferritin-1* and *ferritin-3*) could be used to develop molecular markers for the respective ferritin genes in lentil. In addition, once the lentil whole genome sequence is released cloning and characterization of ferritin and other iron metabolism related genes will be easier.

In gene expression analysis under excess iron it was observed that only samples with 2 h excess iron treatments exhibited significant differential gene expression (**Table 5**) for both genes (*Ferritin-1* and *IRT-1*) in root tissues. The absence of such kinetics in gene expression change for samples that were given 8 or 24 h excess iron treatments across the tissues was observed. The possible reason could be the different iron homeostasis mechanisms in lentil compared to other plant species studied under similar conditions. Development of an assay to find out the reason behind such variation could first start with the standardization of external iron treatments in lentil. In common bean by applying identical excess iron concentration (Lobréaux et al., 1995) in leaf tissue similar kinetics of differential gene expression of ferritin genes (*PvFer1, PvFer2*, and *PvFer1*) were observed (DeLaat et al., 2014). Out of the three genotypes (IAC-Diplomata, Carioca, and BAT 477) used there had been significant genotypic differences of ferritin gene expression for two ferritin genes (PvFer1, PvFer2) (DeLaat et al., 2014). There were no significant differences among the treatments (control with distilled water, osmotic shock causing polyethylene glycol (PEG) treated, excess iron treated, PEG + excess iron treated) for any of the ferritin genes (DeLaat et al., 2014). The interaction between time and treatment was only significant for the PvFer2 and interaction between time and cultivar was significant for the PvFer3 ferritin gene (DeLaat et al., 2014). In most of the treatments ferritin genes were up regulated, however, there were treatments where PvFer1 and PvFer3 were down regulated (DeLaat et al., 2014) over time. The above-mentioned facts for common bean ferritin genes support the results we obtained in the case of *Ferritin-1* and *IRT-1* genes under identical conditions. Further, the gene expression levels for iron metabolism related genes were low in lentil as evident by the high *C*_T_ values. Number of biological replications may be increased to improve power of the test. The difference between seedling and adult plant physiology should be taken into consideration in future experiments. In summary, gene specific markers were developed for three iron metabolism related genes (*Ferritin-1, BHLH-1, IRT-1*) in lentil using PCR based cloning and significant differential expression was observed for *Ferritin-1* and *IRT-1* genes at the transcriptional level.

## Author Contributions

Conceived and designed the experiments: KM, DSG, SK. Performed the experiments: DSG. Analyzed the data: DSG. Contributed reagents/materials/analysis tools: KM. Wrote the paper: DSG, KM, SK. All authors have read and approved the manuscript.

## Conflict of Interest Statement

The authors declare that the research was conducted in the absence of any commercial or financial relationships that could be construed as a potential conflict of interest.

## Footnotes

1 http://knowpulse.usask.ca/portal/blast/nucleotide/nucleotide

2 http://www.ncbi.nlm.nih.gov/tools/primer-blast/
