# Supplementary material for: Development of Molecular Markers for Iron Metabolism Related Genes in Lentil and Their Expression Analysis under Excess Iron Stress
Source: Front Plant Sci. 2017 Apr 13;8:579. doi: 10.3389/fpls.2017.00579 (PMC5390492; doi:10.3389/fpls.2017.00579)
Supplement: Supplementary file 1 [file Table_1.docx]

Table S1. Details of RNA quality data of the 36 samples of *Lens culinaris.*

| **Time course (h)** | **Treatment condition** | **Type of tissue** | **Replication** | **Absorbance at 260/280 nm** | **Absorbance at 260/230 nm** |
| --- | --- | --- | --- | --- | --- |
| 2 | control | shoot | 1 | 2.17 | 2.21 |
| 8 | control | shoot | 2 | 2.19 | 2.38 |
| 24 | control | shoot | 3 | 2.20 | 2.35 |
| 2 | control | shoot | 1 | 2.18 | 2.15 |
| 8 | control | shoot | 2 | 2.16 | 1.85 |
| 24 | control | shoot | 3 | 2.15 | 1.60 |
| 2 | control | shoot | 1 | 2.15 | 1.36 |
| 8 | control | shoot | 2 | 2.19 | 2.05 |
| 24 | control | shoot | 3 | 2.17 | 1.84 |
| 2 | control | root | 1 | 2.16 | 2.08 |
| 8 | control | root | 2 | 2.16 | 2.25 |
| 24 | control | root | 3 | 2.12 | 2.22 |
| 2 | control | root | 1 | 2.18 | 2.30 |
| 8 | control | root | 2 | 2.13 | 2.33 |
| 24 | control | root | 3 | 1.77 | 1.86 |
| 2 | control | root | 1 | 2.14 | 2.64 |
| 8 | control | root | 2 | 2.08 | 3.83 |
| 24 | control | root | 3 | 2.16 | 2.40 |
| 2 | Excess iron | shoot | 1 | 2.18 | 2.47 |
| 8 | Excess iron | shoot | 2 | 2.17 | 2.50 |
| 24 | Excess iron | shoot | 3 | 2.14 | 2.27 |
| 2 | Excess iron | shoot | 1 | 2.09 | 1.88 |
| 8 | Excess iron | shoot | 2 | 2.12 | 2.19 |
| 24 | Excess iron | shoot | 3 | 2.10 | 2.31 |
| 2 | Excess iron | shoot | 1 | 2.11 | 2.30 |
| 8 | Excess iron | shoot | 2 | 2.06 | 1.55 |
| 24 | Excess iron | shoot | 3 | 2.14 | 2.21 |
| 2 | Excess iron | root | 1 | 2.15 | 2.30 |
| 8 | Excess iron | root | 2 | 1.92 | 1.97 |
| 24 | Excess iron | root | 3 | 2.15 | 1.88 |
| 2 | Excess iron | root | 1 | 2.16 | 1.35 |
| 8 | Excess iron | root | 2 | 2.18 | 2.07 |
| 24 | Excess iron | root | 3 | 2.19 | 2.29 |
| 2 | Excess iron | root | 1 | 2.06 | 1.36 |
| 8 | Excess iron | root | 2 | 2.03 | 1.31 |
| 24 | Excess iron | root | 3 | 1.87 | 1.66 |
